# Polymorphisms in *Pvkelch12* and gene amplification of *Pvplasmepsin4* in *Plasmodium vivax* from Thailand, Lao PDR and Cambodia

**DOI:** 10.1186/s12936-019-2749-3

**Published:** 2019-04-02

**Authors:** Jureeporn Duanguppama, Vivek Bhakta Mathema, Rupam Tripura, Nicholas P. J. Day, Mayfong Maxay, Chea Nguon, Lorenz von Seidlein, Mehul Dhorda, Thomas J. Peto, Francois Nosten, Nicholas J. White, Arjen M. Dondorp, Mallika Imwong

**Affiliations:** 1 Department of Molecular Tropical Medicine and Genetics, Faculty of Tropical Medicine, Mahidol University, Bangkok, Thailand; 2 Mahidol-Oxford Tropical Medicine Research Unit, Faculty of Tropical Medicine, Mahidol University, Bangkok, Thailand; 3 Centre for Tropical Medicine, Churchill Hospital, Oxford, UK; 4 Shoklo Malaria Research Unit, Mae Sot, Faculty of Tropical Medicine, Mahidol University, Mae Sot, Thailand; 5 Lao-Oxford-Mahosot Hospital-Wellcome Trust Research Unit, Mahosot Hospital, Vientiane, Lao People’s Democratic Republic; 6 Institute of Research and Education Development, University of Health Sciences, Vientiane, Lao People’s Democratic Republic; 7 National Centre for Parasitology, Entomology & Malaria Control, Ministry of Health, Phnom Penh, Cambodia

**Keywords:** Drug pressure, SNPs, *P. vivax*, CNV, Malaria, Mutations

## Abstract

**Background:**

Mutations in *Pfkelch13* and *Pfplasmepsin2/3* gene amplification are well-established markers for artemisinin and piperaquine resistance in *Plasmodium falciparum,* a widespread problem in the Greater Mekong Subregion (GMS). The *Plasmodium vivax* parasite population has experienced varying drug pressure dependent on local drug policies. We investigated the correlation between drug pressure from artemisinins and piperaquine and mutations in the *P. vivax* orthologous genes *Pvkelch12* and *Pvplasmepsin4* (*Pvpm4*), as candidate resistance markers.

**Methods:**

Blood samples from 734 *P. vivax* patients were obtained from Thailand (n = 399), Lao PDR (n = 296) and Cambodia (n = 39) between 2007 and 2017. *Pvkelch12* and *Pvpm4* was amplified and sequenced to assess gene mutations. To assess *PvPM4* gene amplification, a Taqman^®^ Real-Time PCR method was developed and validated. Selection of non-synonymous mutations was assessed by its ratio with synonymous mutations (Ka/Ks ratios). Mutation rates were compared to the estimated local drug pressure.

**Results:**

Polymorphisms in *Pvkelch12* were rare. *Pvkelch12* mutations V552I, K151Q and M124I were observed in 1.0% (7/734) of *P. vivax* samples. V552I was the most common mutation with a frequency of 0.7% (5/734), most of which (4/5) observed in Ubon Ratchathani, Thailand. Polymorphisms in *Pvpm4* were more common, with a frequency of 40.3% (123/305) in 305 samples from Thailand, Lao PDR and Cambodia, but this was not related to the estimated piperaquine drug pressure in these areas (Pearson’s χ^2^ test, *p* = 0.50). *Pvpm4* mutation V165I was most frequent in Tak, Thailand (40.2%, 43/107) followed by Pailin, Cambodia (43.5%, 37/85), Champasak, Lao PDR (40.4%, 23/57) and Ubon Ratchathani, Thailand (35.7%, 20/56). *Pvpm4* amplification was not observed in 141 samples from Thailand and Cambodia. For both *Pvkelch12* and *Pvpm4*, in all areas and at all time points, the Ka/Ks values were < 1, suggesting no purifying selection.

**Conclusions:**

A novel real-time PCR-based method to assess *P. vivax Pvpm4* gene amplification was developed. Drug pressure with artemisinins and piperaquine in the GMS was not clearly related to signatures of selection for mutations in the *P. vivax* orthologous resistance genes *Pvkelch12* and *Pvpm4* in areas under investigation. Current resistance of *P. vivax* to these drugs is unlikely and additional observations including analysis of associated clinical data from these regions could further clarify current findings.

**Electronic supplementary material:**

The online version of this article (10.1186/s12936-019-2749-3) contains supplementary material, which is available to authorized users.

## Background

Anti-malarial drug resistance is a threat for the elimination of falciparum malaria in the Greater Mekong Subregion (GMS) which includes Cambodia, Lao PDR, Myanmar, Thailand, and Vietnam [1]. For over a decade artemisinin-based combination therapy (ACT) has been the first-line treatment for uncomplicated *Plasmodium falciparum* malaria [2]. The artemisinin-based combinations deployed in the GMS since 2001 include artesunate-mefloquine (AS-MQ), dihydroartemisinin-piperaquine (DHA-PPQ) and artemether-lumefantrine (AL). Cambodia was the first country to deploy AS-MQ in 2000. Treatment failure in falciparum malaria was first reported 4 years later in 2004 [3]. DHA-PPQ was introduced in Cambodia in 2003 and treatment failure was first reported in 2010 [4]. Thailand started implementation of AS-MQ in 1990 and first reported increased treatment failure in 2010. DHA-PPQ was more recently introduced in Thailand and increased treatment failure was recently observed in a northeastern province close to the Cambodian border in 2015–2016 (unpublished). Countries were stratified according to their first-line treatments for *P. vivax* (chloroquine in Lao PDR and Thailand; ASMQ + primaquine in Cambodia), as well as according to the first-line treatment for *P. falciparum* (AL in Lao PDR, DHA-PPQ in Thailand, ASMQ in Cambodia) [1].

Compared to *P. falciparum*, *P. vivax* is geographically the most widespread human malaria with over 2.5 billion people living at risk of infection [5]. Vivax malaria has a high prevalence in South-East Asia, Western Pacific regions, and Central and South America [6–8]. In the GMS the proportion of *P. vivax* and *P. falciparum* cases are estimated as 34% and 66%, respectively [1]. In addition to clinical cases, prevalence of *P. vivax* in asymptomatic parasite carriers, as assessed by PCR methods, is also high, with an average of 30% [9]. In Lao PDR, mixed infections of *P. vivax* and *P. falciparum* account for up to 6.5% of clinical malaria cases [10]. Therefore, the *P. vivax* population will be exposed to ACT used for treatment of falciparum malaria, even where chloroquine is still the first-line drug for *P. vivax* treatment [1]. Drug pressure from ACT on the *P. vivax* parasite population is higher in Cambodia where ACT is the first-line treatment for *P. vivax.*

**Fig. 1:**
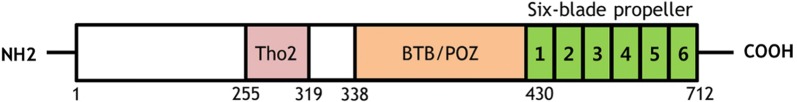
Schematic representation of *Pvkelch12* gene. The *Pvkelch12* is located on chromosome 12 with nucleotide sequence length of 2139 bp which codes for 712 amino acids. The gene itself has been predicted to consist of three domains: Tho2 super family (65 bp), BTB/POZ (93 bp) and Six-blade propeller (283 bp)

Single Nucleotide Polymorphisms (SNPs) at multiple loci in the propeller region of the *P. falciparum kelch* (*Pfkelch13*) gene are the main genetic marker for artemisinin resistance and have reached high frequencies in most countries of the GMS. Examples are the nonsynonymous mutations in *Pfkelch13;* C580Y, R539T and Y439H, with C580Y over time becoming the most dominant mutation [11–15]. The orthologous gene for *Pfkelch13* in *P. vivax* (*Pvkelch12*) has been identified and mapped onto chromosome 12 (accession number PVX_083080, *kelch12* propeller) with a nucleotide and protein sequence length of 2139 bp and 712 amino acids, respectively (Fig. 1). Few *Pvkelch12*-associated nonsynonymous mutations have been identified in *P. vivax* isolates in Cambodia [16]. In addition to artemisinin resistance, countries in the GMS have witnessed more recently the emergence and spread of piperaquine resistant *P. falciparum*. Gene amplification of the *P. falciparum plasmepsin 2* and *3* genes (*Pfplasmepsin2/3*) has been identified as a marker for piperaquine resistance [3, 17, 18]. The orthologous gene for *Pfplasmepsin2/3* in *P. vivax* is *P. vivax plasmepsin4 (Pvpm4),* identified and mapped for *P. vivax* reference strain Sal-1 located on chromosome 13 (accession number PVX_086040). It has a sequence length of 1353 bp and codes for a 450 amino acids protein [16]. Recently genetic variation in *Pvpm4* has been reported from Malaysia, Thailand and Indonesia, in particular the nonsynonymous mutation *Pvplasmepsin4* I165V [19]. There is no information on copy number variation (CNV) in *Pvpm4* in *P. vivax* field isolates.

In the current study, gene polymorphisms in *Pvkelch12* and *Pvpm4* were accessed in *P. vivax* field isolates from three countries of the GMS (Thailand, Lao PDR and Cambodia), and related this to the estimated drug pressure on the parasite population. For this, a real-time PCR method was developed and optimized for *Pvpm4* CNV analysis in field isolates.

## Methods

### Study sites and sample collection

Whole blood samples from both clinical cases and asymptomatic carriers of *P. vivax* were obtained in Thailand (Ubon Ratchathani and Tak), Lao PDR (Champasak, Savannakhet, Xekong and Salavan) and Cambodia (Pailin) between 2007 and 2017. Genomic DNA was extracted from whole blood by using QIAamp^®^ DNA Blood Mini Kit (Qiagen, Germany) and purified DNA was stored at − 20 °C until further use. The study was approved by the Ethics Committee of Faculty of Tropical Medicine, Mahidol University, Thailand (EC approval number MUTM 2017-037-01).

### Amplification and sequencing of *Pvkelch12 and Pvpm4*

Primers designed for *Pvkelch12* and *Pvpm4* genes were based on the *P. vivax* Sal-1 genome (Table 1). DNA of the *Pvkelch12* and *Pvpm4* genes was amplified by semi-nested PCR (Table 1). For PCR amplification the primary and secondary reaction volumes were 25 and 100 µl, respectively. The amplified PCR products were further purified by using PCR purification kit, FavorPrep™ (Favorgen, Taiwan), following standard manufacturer’s instructions. The specificity of all primer pairs designed for this study was tested against both human and non-human primate *Plasmodium* species, and no cross-reactivity was observed with non-*P. vivax* species, to exclude cross-species contamination of PCR products. The nucleotide sequences of *Pvkelch12* and *Pvpm4* genes obtained in this study have been submitted to GenBank database under the accession numbers MK513662-MK513680 and MK513681-MK513709, respectively (Additional file 1).

**Table 1:** Sequence specific oligonucleotide primers, probes and temperature profiles for *Pvkelch12* and *Pvpm4* genes

Gene	Method	Fragment	Primer name	Sequence (5′–3′)	PCR condition			
					Annealing Temperature (°C)	MgCl_2_ (mM)	No. of PCR cycle	Estimated PCR product Size (bp)
*Pvkelch12*	PCR	Nest 1	K12_F1-F	CCATACGTAAACGCTGCAAAT	58	2	25	2139
			K12_c.2216R	CTCCCCATCTGTTCCATGTC				
		F1	K12_F1-F	CCATACGTAAACGCTGCAAAT	58	2	30	980
			K12_F1-R*	TTCTTAATTTGTTTATACCCGTTTGA				
		F2	K12_F2-F	CAAGCTTTTTAAAGACAAAAAGGAA	58	2	30	728
			K12_F2-R*	CAGTTTCGAAAAGGGCTTTATAATC				
		F3	K12_c.1304F*	TGGTTTCGATGGGGTAGAGT	58	2	30	912
			K12_c.2216R	CTCCCCATCTGTTCCATGTC				
*Pvpm4*	PCR	Nest 1	PV_PM4F1_F	TCAAAAGGAGTACGAAGCATACAA	62	2	30	1703
			PV_PM4F2_R	TGTTCTAATTACAGCACCAACACA				
		F1	PV_PM4F1_F*	TCAAAAGGAGTACGAAGCATACAA	62	2	30	807
			PV_PM4F1_R*	ATGGGTTCTAAATCATCAGTGTCA				
		F2	PV_PM4F2_F*	GATGCAGCATTAAAAATCTGTACG	62	2	30	1086
			PV_PM4F2_R	TGTTCTAATTACAGCACCAACACA				
*Pvpm4*	Vector-based primer	*Pvpm4*	CPvPM4-F* CPvPM4-R*	AAAATCGAGAGACCCTATGACAAG ATGGGTTCTAAATCATCAGTGTCA	62	2	35	461
		*Pv β*-*tubulin*	CPvtubulin-F*	AAATTAGGGAAGAATACCCAGACC	62	2	35	451
			CPvtubulin-R*	TCACTTGCACACATCATATTCTTG				
*Pvpm4*	Real-time PCR	*Pvpm4*	Pvpm4_F(CNV) Pvpm4_R(CNV) PvPm4_Probe(CNV)	ACCACCAAAAGTTTATGCTCATC CGTAAGACTTAGACTTGCTAGAGTCG 6FAM-CAAAAAATGCAATAGCAGCGGA-TAMRA	58	–	50	133
		*Pv β*-*tubulin*	PvTubulin_F(CNV) PvTubulin_R2(CNV) PvTubulin_Probe(CNV)	GATAATGAGGCCTTGTATGATATTT AGGAAATCTTAACGAACATGTAACT HEX-TGGTTTCTGCTGCCATGTCAGG-TAMRA	58	–	50	123

### DNA sequence analysis

Purified products from the secondary PCR were subjected to DNA sequencing (Macrogen, Korea). Gene sequences of the PCR amplification products of *Pvkelch12* (accession number: XM_001614165.1, Sal-1; PVX_083080) and *Pvpm4* (accession number: XM_001616821.1, Sal-1; PVX_086040) were confirmed using the NCBI’s Blastn and Blastx programs. Subsequently, gene polymorphisms were assessed by comparison with the reference sequences using BioEdit v7.2.5.

### Identification of signature of selection

The ratio of nonsynonymous site (Ka) to synonymous single nucleotide polymorphisms (Ks) or Ka/Ks ratios was assessed to identify signatures of gene selection in the parasite population, using the online available Ka/Ks Calculator (https://kakscalculator.herokuapp.com/calculate.action). This provides the Jukes-Cantor (JC) and Kimuras-two parameters (K2P) as measures of purifying selection [20, 21]. Differences in categorical variables were examined using Pearson’s Chi squared test.

### Development of real-time PCR to assess *Pvpm4* copy number variation (CNV)

A method to assess *Pvpm4* CNV was developed. For this specific primers for *Pvpm4* and *Pvβ*-*tubulin* (internal control in the real-time PCR reaction) were designed based on the *P. vivax* Sal-1 (PVX_086040) reference gene (Table 1). BLAST analysis of amplified fragments showed a high alignment score of 99% for *Pvpm4* and 91% for *Pvβ*-*tubulin* sequence identity compared to the reference *P. vivax* Sal-1 genome. The regions identified for PCR amplification were highly conserved and specific for *P. vivax.* Primers were labelled with 6-carboxyfluorescein (6-FAM) and 6-carboxytetramethyl-rhodamine (TAMRA) as quencher. CNV was assessed by QuantiTect multiplex PCR NoRox kits (Qiagen, Germany) following standard manufacturer’s instructions. *Pvpm4* plasmids with one and two copy numbers were used as calibrators and positive controls. These plasmids were prepared by cloning of plasmid vectors following standard manufacturer’s instructions (pGEM^®^-T Easy Vector Systems, Promega, USA). Each PCR reaction contained a total volume of 10 µl including 2 µl of genomic DNA as the template. PCR master mix was prepared using 1 × QuantiTect Multiplex (NoRox), 0.4 µM of both forward and reverse primers, and 0.2 µM of TaqMan probe and RNase-free water. The thermocycler (Rotor-Gene Q 5plex System, QIAGEN) profile was: initial denaturation at 95 °C for 15 min, 50 cycles of denaturation at 95 °C for 15 s followed by annealing at 58 °C for 1 min, and final extension at 72 °C for 5 min. The detection threshold cycle (Ct) was calculated by using the 2^−ΔΔCT^ method where, ΔΔCt = (Ct of *Pvpm4 *–Ct of *Pvβ*-*tubulin*) of sample—(Ct of *Pvpm4 *–Ct of *Pvβ*-*tubulin*) of *Pvpm4* plasmid clone. The specificity of the real-time PCR method was validated using isolated human DNA from healthy subjects (n = 50), patients with *P. falciparum* (n = 50) and *P. vivax* (n = 50). This confirmed that the used primers were highly specific to *P. vivax* without any undesired amplification of host DNA or *P. falciparum* DNA. *Pvpm4* gene amplification (2 or more) was called when 2^−ΔΔCt^ exceeded 1.5, with a Ct value acquired after less than 35 cycles.

## Results

### Polymorphisms in *Pvkelch12* and *Pvpm4*

**Fig. 2:**
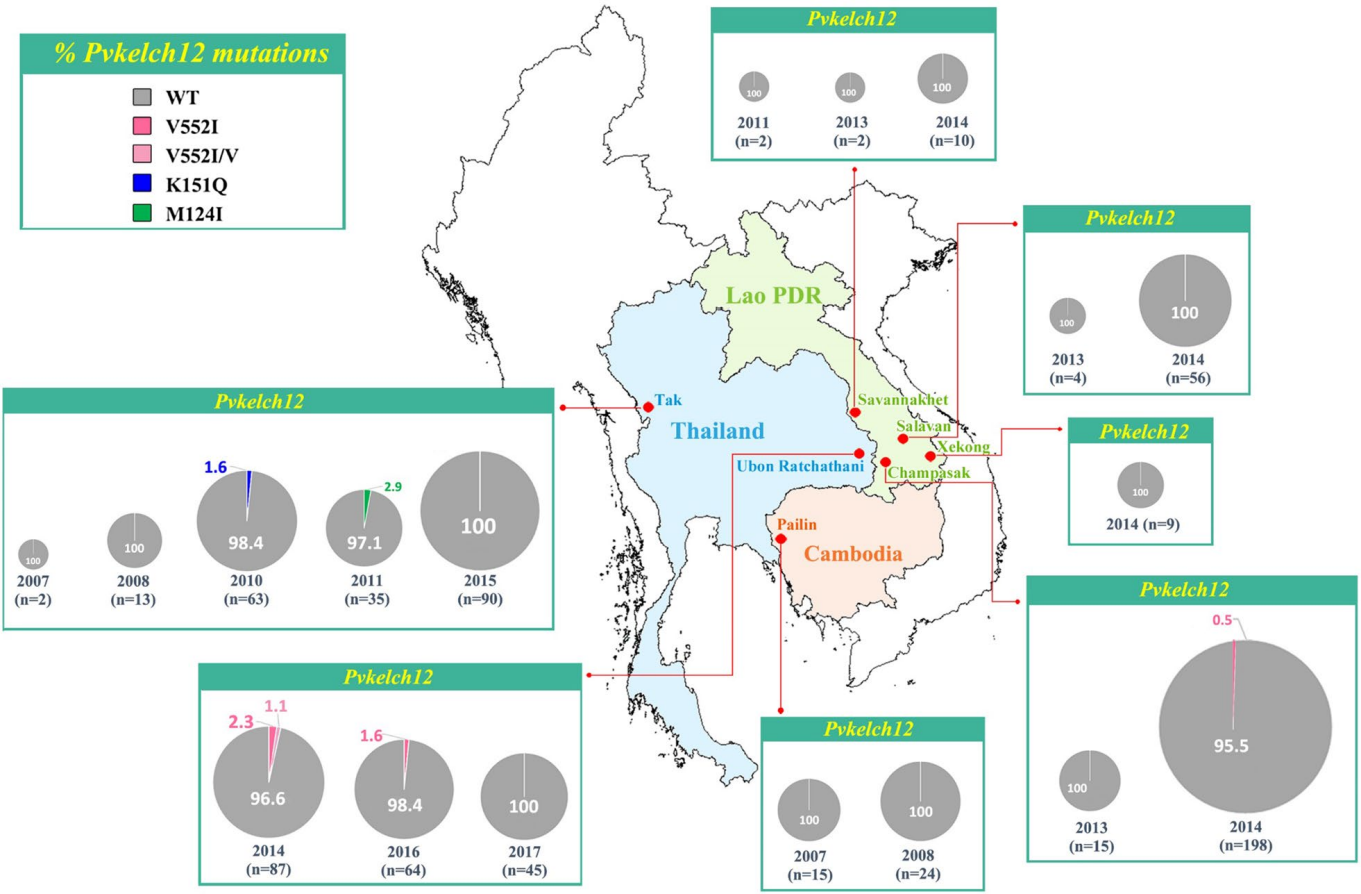
Frequencies of *Pvkelch12* (%) mutations in Thailand, Lao PDR and Cambodia in 2007–2017 (n = 734)

**Fig. 3:**
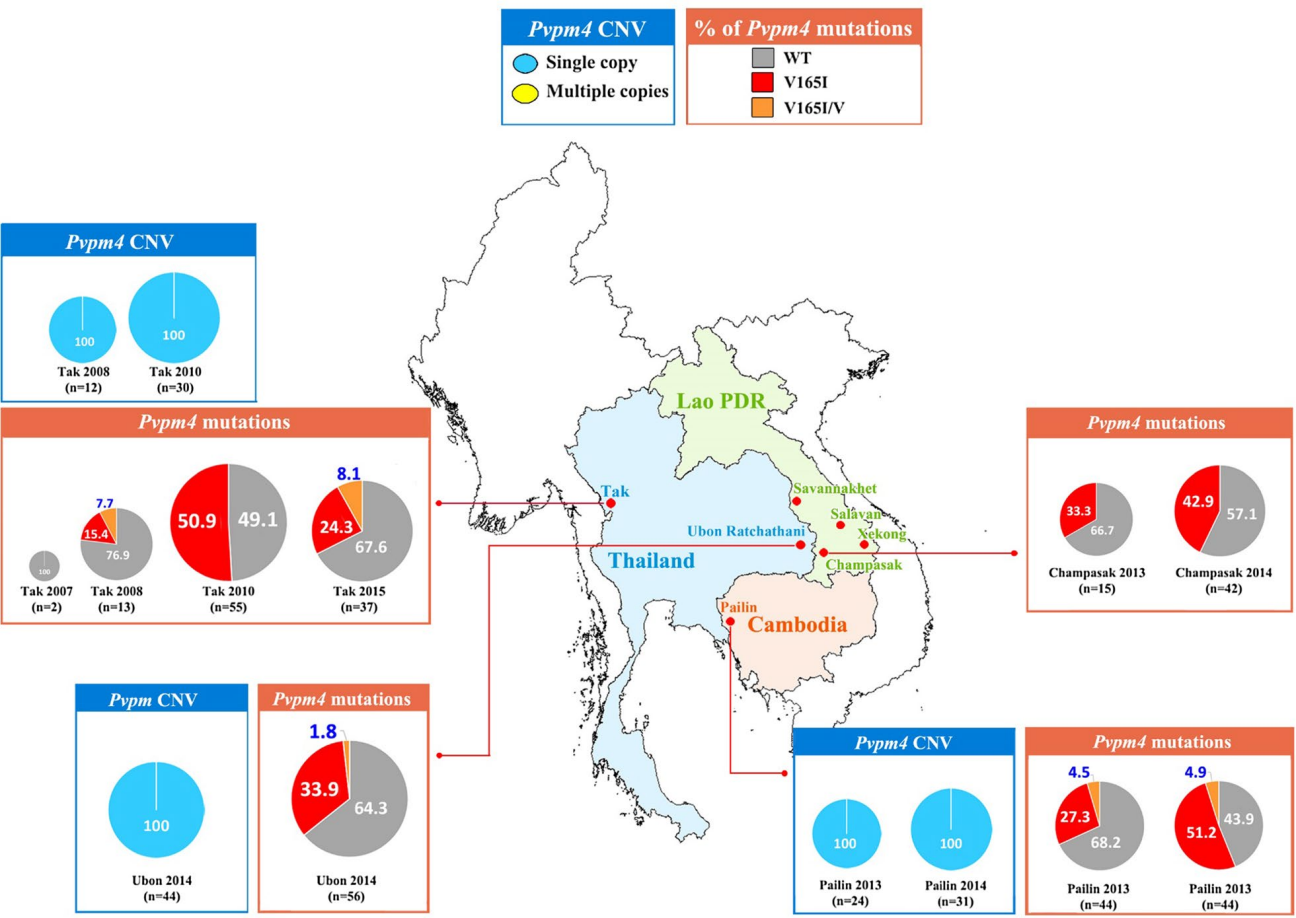
Distribution of *Pvpm4* copy number variations and frequencies of *Pvpm4* (%) mutations in Thailand, Lao PDR and Cambodia in 2007–2017. *Pvpm4* gene amplification was not observed in Ubon Ratchathani, Tak (Thailand) and Pailin (Cambodia) (n = 141)

Analysis of *Pvkelch12* in *P. vivax* strains from Thailand, Lao PDR and Cambodia showed nonsynonymous mutations as 1.0% (7/734) of samples, at codons Val552Ile (V552I), Lys151Gln (K151Q) and Met124Ile (M124I). The V552I mutation was most frequent, followed by K151Q and M124I. V552I has an overall prevalence of 0.7% (5/734) and was observed in Ubon Ratchathani, Thailand (1.4%, 4/286) and Champasak, Lao PDR (0.3%, 1/296). The K151Q and M124I mutations were only observed in Tak, Thailand (0.9%, 1/113 and 0.9%, 1/113) (Fig. 2). Assessment of *Pvpm4* showed nonsynonymous mutation in 40.3% (123/305) of samples, with Val165Ile (V165I), mostly observed in Pailin, Cambodia (43.5%, 37/85) followed by Champasak, Lao PDR (40.4%, 23/57), Tak, Thailand (40.2%, 43/107) and Ubon Ratchathani, Thailand (35.7%, 20/56) (Fig. 3). The observed mutation frequencies in the *Pvkelch12* were not significantly different in regions with ACT as first-line treatment for *P. vivax* (Cambodia) compared to regions with chloroquine as first-line treatment (Thailand and Lao PDR) (Pearson’s χ^2^ test, *p* = 0.38). There was also no association between the frequency of *Pvpm4* gene polymorphisms and deployment of DHA-PPQ (Cambodia versus Thailand and Lao PDR) (Pearson’s χ^2^ test, *p* = 0.50).

### Signatures of selection in *Pvkelch12* and *Pvpm4*

Ratios of nonsynonymous and synonymous mutations were used to identify indicators of selection in *Pvkelch12* and *Pvpm4*. In total, 37/734 (5.1%) samples contained a SNPs in *Pvkelch12*; multiple SNPs in a single strain were not observed. Mutations included three nonsynonymous mutations at codons M124I, K151Q and V552I and ten synonymous mutations at codons N57N, I248I, K310K, I332I, T334T, I340I, S350S, D359D, L365L and T401T (Additional file 2). In *Pvpm4,* 141/305 (46.2%) samples contained four genetic mutations. This included one nonsynonymous mutation at codon V165I and three synonymous mutations at codon Q74Q, G141G and F364F (Additional file 3). The ratios of Ka/Ks for *Pvkelch 12* and *Pvpm4* gene were < 1 for whole sample set, as well as for parasite from the individual countries (Thailand, Lao PDR and Cambodia). This suggests absence of purifying selection in these genes (Tables 2 and 3).

**Table 2:** The ratio of nonsynonymous substitutions per site and synonymous substitutions per site in *Pvkelch12* gene

Countries	Provinces	Year	Total	Ka	Ks	Ka/Ks^a^
Thailand	Tak	2007	2	0	0	Error
	Tak	2008	13	0	0	Error
	Tak	2010	63	3.72 × 10^−3^	5.11 × 10^−3^	0.728
	Tak	2011	35	2.48 × 10^−3^	2.55 × 10^−3^	0.9703
	Ubon Ratchathani	2014	87	2.48 × 10^−3^	5.12 × 10^−3^	0.4839
	Tak	2015	90	0	2.55 × 10^−3^	0
	Ubon Ratchathani	2016	64	2.48 × 10^−3^	2.56 × 10^−3^	0.9682
	Ubon Ratchathani	2017	45	1.24 × 10^−3^	− 1.31815	− 940.45623
Lao PDR	Savannakhet	2011	2	0	0	Error
	Champasak	2013	15	0	0	Error
	Savannakhet	2013	2	0	0	Error
	Salavan	2013	4	0	0	Error
	Champasak	2014	198	2.48 × 10^−3^	5.12 × 10^−3^	0.4839
	Savannakhet	2014	10	1.24 × 10^−3^	− 1.318159	− 940.45623
	Salavan	2014	56	1.24 × 10^−3^	− 1.31815	− 940.45623
	Xekong	2014	9	0	2.55 × 10^−3^	0
Cambodia	Pailin	2007	15	0	0	Error
	Pailin	2008	24	0	0	Error

^a^Ka and Ks were estimated by using approximate method, Jukes-Cantor (JC) and Kimuras-two parameter models: https://kakscalculator.herokuapp.com/calculate.action [21]

**Table 3:** The ratio of nonsynonymous substitutions (Ka) per site and synonymous substitutions (Ks) per site in *Pvpm4 gene*

Frequence of *Pfplasmepsin2 CNV*	Treatment guidelines for *P. falciparum*	Location	Year	Total	Ka	Ks	Ka/Ks^a^
*Pfplasmepsin2* (< 5%)	Artesunate-Mefloquine	Thailand (Tak)	2007–2012	107	2.04 × 10^−3^	8.22 × 10^−3^	0.2488
	DHA-PPQ	Thailand (Ubon Ratchathani)	2012–2015	56	2.04 × 10^−3^	4.12 × 10^−3^	0.4968
	Artemether–lumefantrine	Lao PDR (Champasak)	2013–2014	57	2.04 × 10^−3^	4.10 × 10^−3^	0.4985
*Pfplasmepsin2* (5–30%)	DHA-PPQ	Cambodia (Pailin)	2013–2014	85	2.04 × 10^−3^	0	Error

^**a**^Ka/Ks ratios of *Pvpm4* are grouped by presumed DHA-PPQ drug pressure based on *Pfplasmepsin2* amplification prevalence in *P. falciparum* in the same areas [33, 34]. Ka and Ks were estimated by using approximate method, Jukes-Cantor (JC) and Kimuras-two parameter models: https://kakscalculator.herokuapp.com/calculate.action [21]

### *Pvpm4* copy number variation

CNV in *Pvpm4* was assessed in 141 *P. vivax* samples from Ubon Ratchathani, Tak (Thailand) and Pailin (Cambodia) obtained between 2008 and 2014. Gene amplification in *Pvpm4* was not observed in any sample under investigation (Fig. 3). Details of the assessment are provided in Additional file 4.

## Discussion

Resistance in *P. falciparum* to both artemisinin and piperaquine is now widespread in Cambodia, northeastern Thailand and Southern Lao PDR, and genetic epidemiological studies have identified that this has spread from a single parasite lineage emerging from western Cambodia [15]. In Cambodia, similar drug pressure from DHA-PPQ on the *P. vivax* as on the *P. falciparum* parasite population can be expected, since DHA-PPQ is also first-line treatment for vivax malaria in this country [1], and because of the frequent co-infection with the two *Plasmodium* species [9]. Thus, vigilance is warranted for surveillance of resistance in *P. vivax* towards these drugs. This surveillance is hampered by the difficulty to perform therapeutic efficacy studies in vivax malaria, since recrudescent infections denoting drug resistance are difficult to distinguish from relapse infections from liver hypnozoites. Also, compared to *P. falciparum*, methods for in vitro drug sensitivity testing for artemisinins and piperaquine are not well established in *P. vivax*. Molecular markers for drug resistance in *P. vivax* would facilitate surveillance, but no markers have been identified to date. A first step for identifying novel markers would be to study orthologous resistance genes of *P. falciparum* in *P. vivax*, and establish whether mutations in these genes are selected for according to the level of drug pressure. The current study focused on *Pvkelch* propeller region mutations as a putative marker for artemisinin resistance and *Pvpm4* mutations and amplification for piperaquine resistance.

The family of *Plasmodium* KELCH proteins consist of six KELCH motifs in the propeller domain, linked through a Broad-Complex Tramtrack and Bric-a-Brac/Pox virus and Zinc finger (BTB/POZ) domain, to the transcription factor/nuclear export subunit protein 2 (Tho2) domain. The *kelch* gene is highly conserved across different *Plasmodium* species, with a ratio between nonsynonymous and synonymous mutations (Ka/Ks ratio) around 1 in drug sensitive *Plasmodium* populations [22, 23]. SNPs in the propeller regions of *Pfkelch13* are closely linked to the slow clearance *P. falciparum* phenotype denoting artemisinin resistance. Although only single mutation are commonly observed in the propeller region of *Pfkelch13* gene, study has shown that multiple mutations are also tolerated [14]. Selection of *Pfkelch13* mutations in areas of artemisinin resistance results in a Ka/Ks ratio of around 10^2^ in countries in the GMS [11, 23].

In Africa, nonsynonymous mutations in *Pfkelch13* are observed in low frequencies (few percent), without signatures of selection, likely denoting a background mutation rate in the gene [24]. Overall mutation rates for *P. falciparum* are in the range of 1–10 × 10^−9^ per nucleotide site per mitotic division [25–27]. Depending on the fitness loss induced by the mutation, subsequent drug pressure can select for the drug resistant gene, increasing its frequency above background and increasing the Ka/Ks ratio [25]. Present study indicates that only 0.95% of parasites harbored nonsynonymous mutations in *Pvkelch12*, whereas synonymous mutations were observed in 4.1% of parasites. This was not different between countries. These findings are in agreement with a previous report showing limited genetic diversity of *Pvkelch12* in Southeast Asia [28]. Both the low frequency and the low Ka/Ks ratio suggest that there is currently no selection of *Pvkelch12* mutations in *P. vivax* in the study areas in Thailand, Cambodia and Lao PDR, despite the widespread exposure to ACT. Although it is not clearly understood whether *Pvkelch12* plays a role in *P. vivax* resistance to artemisinins, absence of *Pvkelch12* mutations corroborates the absence of clinical resistance to artemisinins in these areas.

Of the *Pvkelch12* mutations identified during current study, mutations at position V552I and M124I have been reported previously (Additional file 5); the G581R mutation previously reported from China was not observed in the current study [28–30]. The orthologous *Pfkelch13* mutations at positions 555, 126 and 595, have not been described in association with artemisinin resistance in falciparum malaria. Vice versa, none of the currently validated artemisinin associated *Pfkelch13* mutations have to date been described in the orthologous positions in *Pvkelch12* [31].

*Plasmepsins I, II, III, and IV* in *Plasmodium* constitute a group of histo-aspartic proteases aiding the hydrolysis of haemoglobin in the plasmodial food vacuole, providing amino acids as food for the erythrocytic stage of *P. falciparum*. Gene amplification of *Pfplasmepsin2* and *Pfplasmepsin3* are strongly associated with resistance to piperaquine in falciparum malaria. A potential mechanism is interference with the mechanism of action of this drug, which is inhibiting conversion of toxic haem moieties to nontoxic haemozoin during haemoglobin digestion [32]. Frequencies of CNV in the *P. vivax* orthologous gene *Pvpm4* have not been studied before, and a method for assessing *Pvpm4* copy numbers was developed for the purpose of this study. However, *Pvpm4* gene amplification was not observed in any of the *P. vivax* samples from Thailand and Cambodia. In contrast, SNPs in *Pvpm4* were frequently observed, in particular the nonsynonymous mutation V165I (123/305 or 40.3% of samples). The mutation has been described previously in *P. vivax* from Malaysia (Sabah) and Thailand [19]. However, it is unlikely that this mutation is related to piperaquine drug resistance, since its frequency was not associated with the level of piperaquine drug pressure (low in Thailand, high in Cambodia) (Table 3).

Although the current study did not show signatures of selection for the identified mutations in *Pvkelch12* and *Pvpm4*, it is warranted to continue monitor these mutations over time and over a larger geographical area, including Vietnam and Myanmar. In addition, future studies on potential molecular markers for resistance in *P. vivax* can include associations with the clinical phenotype (parasite clearance rate and treatment failure) and protein level analysis of *Pvkelch12* and *Pvpm4.*

## Conclusions

Present study assessed mutations in the *P. vivax* orthologous resistance genes *Pvkelch12* for artemisinin resistance and *Pvpm4* for piperaquine resistance. Although nonsynonymous mutations were observed, the frequency was low, and not higher than synonymous mutations in the same genes. *Pvpm4* gene amplification was not observed. Current resistance of *P. vivax* in Thailand, Lao PDR and Cambodia to these drugs is unlikely. Nonetheless, additional observations, validations and analysis of associated clinical data from these malaria endemic zones could further help clarify current findings.

## Additional files

**Additional file 1.** Accession numbers of nucleotide sequences and sequence IDs of *Pvkelch12* and *Pvpm4* genes obtained from GenBank database.

**Additional file 2.** Synonymous mutations in *Pvkelch12* gene Thailand, Lao PDR and Cambodia.

**Additional file 3.** Synonymous mutations in *Pvpm4* gene Thailand, Lao PDR and Cambodia.

**Additional file 4.** The distribution of *Pvpm4* copy number variations in each sample from 3 study sites with geometric means with 95% confidence interval of *Pvpm4* CNV.

**Additional file 5.** Comparison of *Pvkelch12* mutations of this study to previous publications.

## Abbreviations

*Pvkelch12*: *P. vivax kelch*
*Pvpm4*: *P. vivax plasmepsin4*
SNPs: single nucleotide polymorphisms
CNV: copy number variation
qPCR: quantitative real-time PCR
