# Supplementary material for: Polymorphisms in Pvkelch12 and gene amplification of Pvplasmepsin4 in Plasmodium vivax from Thailand, Lao PDR and Cambodia
Source: Malar J. 2019 Apr 2;18:114. doi: 10.1186/s12936-019-2749-3 (PMC6444602; doi:10.1186/s12936-019-2749-3)
Supplement: Supplementary file 1 — Additional file 1. Accession numbers of nucleotide sequences and sequence IDs of Pvkelch12 and Pvpm4 genes obtained from GenBank database. [file 12936_2019_2749_MOESM1_ESM.docx]

**Additional file 1: Accession numbers of nucleotide sequences and sequence IDs of *Pvkelch12* and *Pvpm4* genes obtained from GenBank database**

| **No.** | **Gene** | **Sequence IDs** | **Collection date** | **Country** | **Isolation source** | **Nucleotide (amino acid) change *** | **Accession numbers** |
| --- | --- | --- | --- | --- | --- | --- | --- |
| 1 | *Pvpm4* | Sq01PvThaiT01A | 2007 | Thailand | Tak | Wild type | MK513662 |
| 2 | *Pvpm4* | Sq02PvThaiT02A | 2008 | Thailand | Tak | Wild type | MK513663 |
| 3 | *Pvpm4* | Sq04PvThaiT04A | 2010 | Thailand | Tak | Wild type | MK513664 |
| 4 | *Pvpm4* | Sq06PvThaiT06A | 2015 | Thailand | Tak | Wild type | MK513665 |
| 5 | *Pvpm4* | Sq09PvThaiT09A | 2014 | Thailand | Ubon Ratchathani | Wild type | MK513666 |
| 6 | *Pvpm4* | Sq12PvLaoL12A | 2013 | Lao PDR | Champasak | Wild type | MK513667 |
| 7 | *Pvpm4* | Sq14PvLaoL14A | 2014 | Lao PDR | Champasak | Wild type | MK513668 |
| 8 | *Pvpm4* | Sq16PvCambodiaC16A | 2013 | Cambodia | Pailin | Wild type | MK513669 |
| 9 | *Pvpm4* | Sq18PvCambodiaC18A | 2014 | Cambodia | Pailin | Wild type | MK513670 |
| 10 | *Pvpm4* | Sq03PvThaiT03B | 2008 | Thailand | Tak | 493GTC>ATC (V165I) | MK513671 |
| 11 | *Pvpm4* | Sq17PvCambodiaC17B | 2013 | Cambodia | Pailin | 493GTC>ATC (V165I) | MK513672 |
| 12 | *Pvpm4* | Sq19PvCambodiaC19B | 2014 | Cambodia | Pailin | 493GTC>ATC (V165I) | MK513673 |
| 13 | *Pvpm4* | Sq05PvThaiT05C | 2010 | Thailand | Tak | 222CAA>CAG (Q74Q), 423GGA>GGG (G141G), 493GTC>ATC (V165I) | MK513674 |
| 14 | *Pvpm4* | Sq07PvThaiT07D | 2015 | Thailand | Tak | 222CAA>CAG (Q74Q) | MK513675 |
| 15 | *Pvpm4* | Sq08PvThaiT08E | 2015 | Thailand | Tak | 222CAA>CAG (Q74Q), 493GTC>ATC (V165I) | MK513676 |
| 16 | *Pvpm4* | Sq10PvThaiT10F | 2014 | Thailand | Ubon Ratchathani | 423GGA>GGG (G141G), 493GTC>ATC (V165I) | MK513677 |
| 17 | *Pvpm4* | Sq11PvThaiT11G | 2014 | Thailand | Ubon Ratchathani | 423GGA>GGG (G141G) | MK513678 |
| 18 | *Pvpm4* | Sq13PvLaoL13H | 2013 | Lao PDR | Champasak | 493GTC>ATC (V165I), 1092TTC>TTT (F364F) | MK513679 |
| 19 | *Pvpm4* | Sq15PvLaoL15H | 2014 | Lao PDR | Champasak | 493GTC>ATC (V165I), 1092TTC>TTT (F364F) | MK513680 |
| 20 | *Pvkelch12* | Sq20PvThaiT20A | 2007 | Thailand | Tak | Wild type | MK513681 |
| 21 | *Pvkelch12* | Sq21PvThaiT21A | 2008 | Thailand | Tak | Wild type | MK513682 |
| 22 | *Pvkelch12* | Sq22PvThaiT22A | 2010 | Thailand | Tak | Wild type | MK513683 |
| 23 | *Pvkelch12* | Sq24PvThaiT24A | 2011 | Thailand | Tak | Wild type | MK513684 |
| 24 | *Pvkelch12* | Sq26PvThaiT26A | 2015 | Thailand | Tak | Wild type | MK513685 |
| 25 | *Pvkelch12* | Sq28PvThaiU28A | 2014 | Thailand | Ubon Ratchathani | Wild type | MK513686 |
| 26 | *Pvkelch12* | Sq31PvThaiU31A | 2016 | Thailand | Ubon Ratchathani | Wild type | MK513687 |
| 27 | *Pvkelch12* | Sq33PvThaiU33A | 2017 | Thailand | Ubon Ratchathani | Wild type | MK513688 |
| 28 | *Pvkelch12* | Sq35PvLaoL35A | 2011 | Lao PDR | Savannakhet | Wild type | MK513689 |
| 29 | *Pvkelch12* | Sq36PvLaoL36A | 2013 | Lao PDR | Savannakhet | Wild type | MK513690 |
| 30 | *Pvkelch12* | Sq37PvLaoL37A | 2014 | Lao PDR | Savannakhet | Wild type | MK513691 |
| 31 | *Pvkelch12* | Sq39PvLaoL39A | 2013 | Lao PDR | Salavan | Wild type | MK513692 |
| 32 | *Pvkelch12* | Sq40PvLaoL40A | 2014 | Lao PDR | Salavan | Wild type | MK513693 |
| 33 | *Pvkelch12* | Sq42PvLaoL42A | 2014 | Lao PDR | Xekong | Wild type | MK513694 |
| 34 | *Pvkelch12* | Sq44PvLaoL44A | 2013 | Lao PDR | Champasak | Wild type | MK513695 |
| 35 | *Pvkelch12* | Sq45PvLaoL45A | 2014 | Lao PDR | Champasak | Wild type | MK513696 |
| 36 | *Pvkelch12* | Sq47PvCambodiaC47A | 2007 | Cambodia | Pailin | Wild type | MK513697 |
| 37 | *Pvkelch12* | Sq48PvCambodiaC48A | 2008 | Cambodia | Pailin | Wild type | MK513698 |
| 38 | *Pvkelch12* | Sq23PvThaiT23B | 2010 | Thailand | Tak | 451AAG>CAG (K151Q), 1020ATA>ATT (I340I), 1077GAC>GAT (D359D), 1093TTG>CTG (L365L), 1203ACC>ACA (T401T) | MK513699 |
| 39 | *Pvkelch12* | Sq25PvThaiT25C | 2011 | Thailand | Tak | 372ATG>ATA (M124I), 1020ATA>ATT (I340I), 1050TCT>TCA (S350S ) | MK513700 |
| 40 | *Pvkelch12* | Sq27PvThaiT27D | 2015 | Thailand | Tak | 930AAG>AAA (K310K) | MK513701 |
| 41 | *Pvkelch12* | Sq29PvThaiU29E | 2014 | Thailand | Ubon Ratchathani | 171AAC>AAT (N57N), 1002ACG>ACA (T334T), 1020ATA>ATT (I340I) | MK513702 |
| 42 | *Pvkelch12* | Sq30PvThaiU30F | 2014 | Thailand | Ubon Ratchathani | 171AAC>AAT (N57N), 1002ACG>ACA (T334T), 1020ATA>ATT (I340I), 1654GTA>ATA (V552I) | MK513703 |
| 43 | *Pvkelch12* | Sq32PvThaiU32G | 2016 | Thailand | Ubon Ratchathani | 171AAC>AAT (N57N), 1020ATA >ATT (I340I), 1654GTA>ATA (V552I) | MK513704 |
| 44 | *Pvkelch12* | Sq34PvThaiU34H | 2017 | Thailand | Ubon Ratchathani | 1020ATA>ATT (I340I) | MK513705 |
| 45 | *Pvkelch12* | Sq46PvLaoL46I | 2014 | Lao PDR | Champasak | 171AAC>AAT (N57N), 744ATA>ATT (I248I), 996ATC>ATA (I332I), 1002ACG>ACA (T334T), 1020ATA>ATT (I340I), 1654GTA>ATA (V552I) | MK513706 |
| 46 | *Pvkelch12* | Sq38PvLaoL38J | 2014 | Lao PDR | Savannakhet | 744ATA>ATT (I248I) | MK513707 |
| 47 | *Pvkelch12* | Sq41PvLaoL41J | 2014 | Lao PDR | Salavan | 744ATA>ATT (I248I) | MK513708 |
| 48 | *Pvkelch12* | Sq43PvLaoL43K | 2014 | Lao PDR | Xekong | 171AAC>AAT (N57N) | MK513709 |
| * XM_001616821.1 and XM_001614165.1 use as a reference sequences for *Pvpm4* and *Pvkelch12*, respectively | | | | | | |  |
