# Supplementary material for: Polymorphisms in Pvkelch12 and gene amplification of Pvplasmepsin4 in Plasmodium vivax from Thailand, Lao PDR and Cambodia
Source: Malar J. 2019 Apr 2;18:114. doi: 10.1186/s12936-019-2749-3 (PMC6444602; doi:10.1186/s12936-019-2749-3)
Supplement: Supplementary file 2 — Additional file 2. Synonymous mutations in Pvkelch12 gene Thailand, Lao PDR and Cambodia. [file 12936_2019_2749_MOESM2_ESM.docx]

**Additional file 2: Synonymous mutations of *Pvkelch12* gene in Thailand, Lao PDR and Cambodia**

| **Country** | **Location** | **Year** | **Total** | **Synonymous mutations (Reference : *P. vivax kelch* gene (PVX_083080)** | | | | | | | | | |
| --- | --- | --- | --- | --- | --- | --- | --- | --- | --- | --- | --- | --- | --- |
|  |  |  |  | ***Pfkelch13*** | | | | | | | | | |
|  |  |  |  | **N59** | **L262** | **K324** | **I346** | **T348** | **I354** | **S364** | **D373** | **L379** | **T415** |
|  |  |  |  | ***Pvkelch12*** | | | | | | | | | |
|  |  |  |  | **N57N** | **I248I** | **K310K** | **I332I** | **T334T** | **I340I** | **S350S** | **D359D** | **L365L** | **T401T** |
| Thailand | Tak | 2007 | 2 | 0 | 0 | 0 | 0 | 0 | 0 | 0 | 0 | 0 | 0 |
|  | Tak | 2008 | 13 | 0 | 0 | 0 | 0 | 0 | 0 | 0 | 0 | 0 | 0 |
|  | Tak | 2010 | 63 | 0 | 0 | 0 | 0 | 0 | 3.2% (2/63) | 0 | 1.6% (1/63) | 1.6% (1/63) | 1.6% (1/63) |
|  | Tak | 2011 | 35 | 0 | 0 | 0 | 0 | 0 | 2.9% (1/35) | 2.9% (1/35) | 0 | 0 | 0 |
|  | Tak | 2015 | 90 | 0 | 0 | 1.11% (1/90) | 0 | 0 | 0 | 0 | 0 | 0 | 0 |
|  | Ubon Ratchathani | 2014 | 87 | 3.4% (3/87) | 0 | 0 | 0 | 2.3% (2/87) | 2.3% (2/87) | 0 | 0 | 0 | 0 |
|  | Ubon Ratchathani | 2016 | 64 | 1.6% (1/64) | 0 | 0 | 0 | 0 | 1.6% (1/64) | 0 | 0 | 0 | 0 |
|  | Ubon Ratchathani | 2017 | 45 | 0 | 0 | 0 | 0 | 0 | 4.4% (2/45) | 0 | 0 | 0 | 0 |
| Lao PDR | Savannakhet | 2011 | 2 | 0 | 0 | 0 | 0 | 0 | 0 | 0 | 0 | 0 | 0 |
|  | Champasak | 2013 | 15 | 0 | 0 | 0 | 0 | 0 | 0 | 0 | 0 | 0 | 0 |
|  | Savannakhet | 2013 | 2 | 0 | 0 | 0 | 0 | 0 | 0 | 0 | 0 | 0 | 0 |
|  | Salavan | 2013 | 4 | 0 | 0 | 0 | 0 | 0 | 0 | 0 | 0 | 0 | 0 |
|  | Champasak | 2014 | 198 | 0.5% (1/198) | 0.5% (1/198) | 0 | 0.5% (1/198) | 0.5% (1/198) | 1.0% (2/198) | 0 | 0 | 0 | 0 |
|  | Savannakhet | 2014 | 10 | 0 | 10.0% (1/10) | 0 | 0 | 0 | 0 | 0 | 0 | 0 | 0 |
|  | Salavan | 2014 | 56 | 0 | 5.4% (3/56) | 0 | 0 | 0 | 0 | 0 | 0 | 0 | 0 |
|  | Xekong | 2014 | 9 | 11.1% (1/9) | 0 | 0 | 0 | 0 | 0 | 0 | 0 | 0 | 0 |
| Cambodia | Pailin | 2007 | 15 | 0 | 0 | 0 | 0 | 0 | 0 | 0 | 0 | 0 | 0 |
|  | Pailin | 2008 | 24 | 0 | 0 | 0 | 0 | 0 | 0 | 0 | 0 | 0 | 0 |
|  |  | **Total** | **734** |  |  |  |  |  |  |  |  |  |  |
