# Supplementary material for: Polymorphisms in Pvkelch12 and gene amplification of Pvplasmepsin4 in Plasmodium vivax from Thailand, Lao PDR and Cambodia
Source: Malar J. 2019 Apr 2;18:114. doi: 10.1186/s12936-019-2749-3 (PMC6444602; doi:10.1186/s12936-019-2749-3)
Supplement: Supplementary file 3 — Additional file 3. Synonymous mutations in Pvpm4 gene Thailand, Lao PDR and Cambodia. [file 12936_2019_2749_MOESM3_ESM.docx]

**Additional file 3: Synonymous mutations of *Pvpm4* gene in Thailand, Lao PDR and Cambodia**

| **Countries** | **Provinces** | **Year** | **Total** | **Synonymous mutations** | | |  |
| --- | --- | --- | --- | --- | --- | --- | --- |
|  |  |  |  | **Reference : *Pvpm4* gene (PVX_086040)** | | |  |
|  |  |  |  | ***Pfpm2*** | | |  |
|  |  |  |  | **K75** | **G142** | **F365** |  |
|  |  |  |  | ***Pvpm4*** | | |  |
|  |  |  |  | **Q74Q** | **G141G** | **F364F** |  |
| Thailand | Tak | 2007 | 2 | 0 | 0 | 0 |  |
|  | Tak | 2008 | 13 | 0 | 0 | 0 |  |
|  | Tak | 2010 | 55 | 7.3% (4/55) | 5.5% (3/55) | 0 |  |
|  | Tak | 2015 | 37 | 13.5% (5/37) | 0 | 0 |  |
|  | Ubon Ratchathani | 2014 | 56 | 0 | 7.1% (4/56) | 0 |  |
| Lao PDR | Champasak | 2013 | 15 | 0 | 0 | 6.7% (1/15) |  |
|  | Champasak | 2014 | 42 | 0 | 0 | 2.4% (1/42) |  |
| Cambodia | Pailin | 2013 | 44 | 0 | 0 | 0 |  |
|  | Pailin | 2014 | 41 | 0 | 0 | 0 |  |
|  |  | Total | **305** |  |  |  |  |
