# Supplementary material for: Polymorphisms in Pvkelch12 and gene amplification of Pvplasmepsin4 in Plasmodium vivax from Thailand, Lao PDR and Cambodia
Source: Malar J. 2019 Apr 2;18:114. doi: 10.1186/s12936-019-2749-3 (PMC6444602; doi:10.1186/s12936-019-2749-3)

**Additional file 4: The distribution of *Pvpm4* copy number variations in each sample from 3 study sites with geometric means with 95% confidence interval of *Pvpm4* CNV**


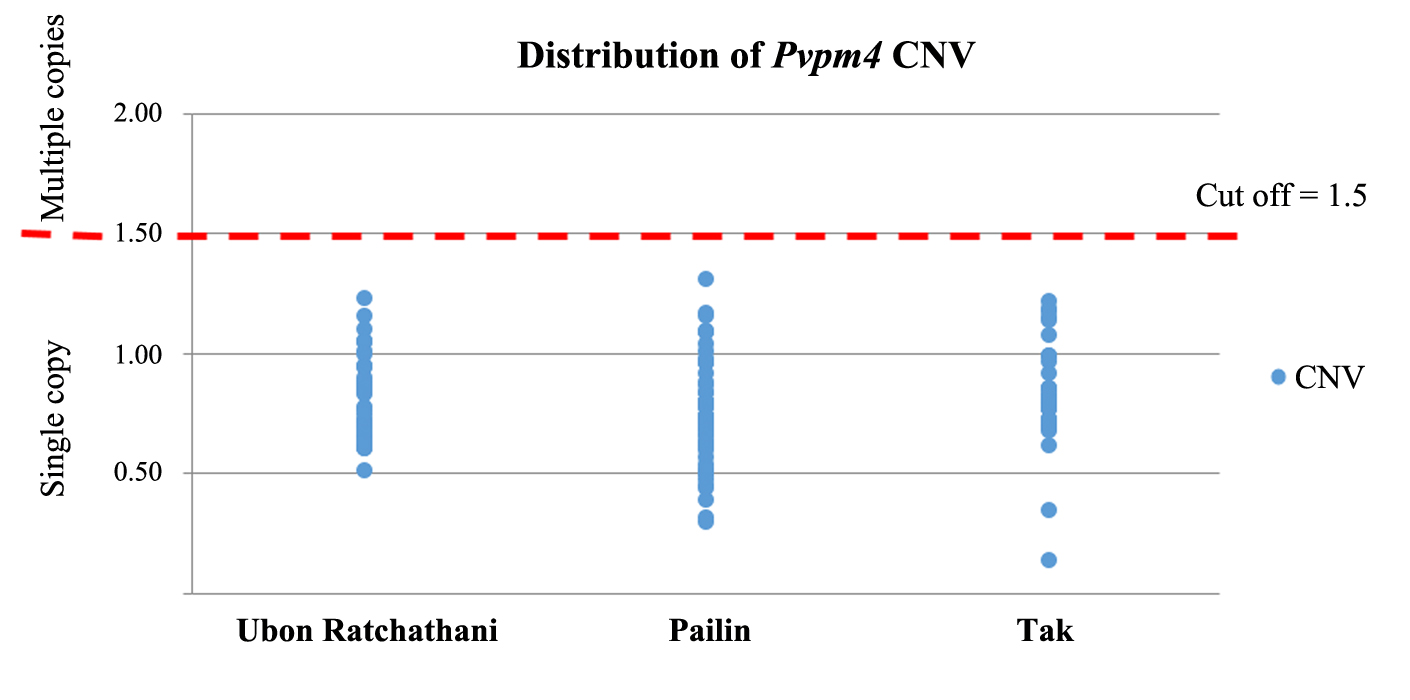

Supplement: Supplementary file 4 — Additional file 4. The distribution of Pvpm4 copy number variations in each sample from 3 study sites with geometric means with 95% confidence interval of Pvpm4 CNV. [file 12936_2019_2749_MOESM4_ESM.docx]
