# Supplementary material for: Polymorphisms in Pvkelch12 and gene amplification of Pvplasmepsin4 in Plasmodium vivax from Thailand, Lao PDR and Cambodia
Source: Malar J. 2019 Apr 2;18:114. doi: 10.1186/s12936-019-2749-3 (PMC6444602; doi:10.1186/s12936-019-2749-3)
Supplement: Supplementary file 5 — Additional file 5. Comparison of Pvkelch12 mutations of this study to previous publications. [file 12936_2019_2749_MOESM5_ESM.docx]

**Additional file 5: Comparison of *Pvkelch12* mutations of this study to previous publications**

| **Countries** | **Year** | **Non-synonymous mutations** | | | | **Synonymous mutations** | | | | | | | | | | | | | | **References** |
| --- | --- | --- | --- | --- | --- | --- | --- | --- | --- | --- | --- | --- | --- | --- | --- | --- | --- | --- | --- | --- |
|  |  | ***Pfkelch13*** | | | | ***Pfkelch13*** | | | | | | | | | | | | | |  |
|  |  | **T126** | **K189T/N** | **V555** | **G595** | **N59** | **N186** | **L262** | **K324** | **I346** | **T348** | **I354** | **S364** | **D373** | **S374** | **L379** | **T415** | **S711** | **G718** |  |
|  |  | ***Pvkelch12*** | | | | ***Pvkelch12*** | | | | | | | | | | | | | |  |
|  |  | **M124I** | **K151Q** | **V552I/V** | **G581R** | **N57N** | **N172N** | **I248I** | **K310K** | **I332I** | **T334T** | **I340I** | **S350S** | **D359D** | **S360S** | **L365L** | **T401T** | **S697S** | **G704G** |  |
| Cambodia* | 2007-2008 | - | - | - | - | - | - | - | - | - | - | - | - | - | - | - | - | - | - | Brazeau et al., 2016 |
| Cambodia | 2011-2013 | - | - | 2/284 (0.7%) | - | - | - | - | - | - | - | - | - | - | - | - | - | - | - | Popovici et al., 2015 |
| China | 2004-2006 | - | - | - | - | - | - | - | - | - | - | - | - | - | - | - | - | - | 1/66 (1.5%) | Wang et al., 2016 |
| China | 2012-2013 | - | - | - | 2/66 (3.0%) | - | - | - | - | - | - | - | - | - | - | - | - | - | - | Wang et al., 2016 |
| Myanmar | 2012-2013 | - | - | - | 1/32 (3.1%) | - | - | - | - | - | - | - | - | - | - | - | - | - | - | Wang et al., 2016 |
| China | 2012-2013 | 2/100 (2.0%) | - | - | - | - | 2/100 (2.0%) | - | - | - | - | - | - | - | 2/100 (2.0%) | - | - | - | - | Deng et al., 2016 |
| Myanmar | 2015 | - | - | - | - | - | - | - | - | - | - | - | - | - | - | - | - | 5/162 (3.0%) | - | Deng et al., 2016 |
| Lao PDR | 2014 | - | - | 1/198 (0.5%) | - | 2/198 (1.0%) | - | 5/198 (2.5%) | - | 1/198 (0.5%) | 1/198 (0.5%) | 2/198 (1.0%) | - | - | 2/198 (1.0%) | - | - | - | - | This study |
| Thailand | 2010 | - | 1/63 (1.6%) | - | - | - | - | - | - | - | - | 2/63 (3.2%) | - | 1/63 (1.6%) | - | 1/63 (1.6%) | 1/63 (1.6%) | - | - | This study |
| Thailand | 2011 | 1/35 (2.9%) | - | - | - | - | - | - | - | - | - | 1/35 (2.9%) | 1/35 (2.9%) | - | - | - | - | - | - | This study |
| Thailand | 2014 | - | - | 3/87 (3.4%) | - | 3/87 (3.4%) | - | - | - | - | 2/87 (2.3%) | 2/87 (2.3%) | - | - | - | - | - | - | - | This study |
| Thailand | 2015 | - | - | - | - | - | - | - | 1/90 (1.1%) | - | - | - | - | - | - | - | - | - | - | This study |
| Thailand | 2016 | - | - | 1/64 (1.6%) | - | 1/64 (1.6%) | - | - | - | - | - | 1/64 (1.6%) | - | - | - | - | - | - | - | This study |
| Thailand | 2017 | - | - | - | - | - | - | - | - | - | - | 2/45 (4.4%) | - | - | - | - | - | - | - | This study |

*There is no mutation in 39 samples from Cambodia. Apart from whole gene sequencing, *Pvkelch12* mutations was also detected in Cambodia though next generation sequencing and found both non-synonymous and synonymous mutation as follow; G23E, I248I, I340I, V552I, D627D and C682C (Brazeau et al., 2016)
